# Validation of a yellow fever vaccine model using data from primary vaccination in children and adults, re-vaccination and dose-response in adults and studies with immunocompromised individuals

**DOI:** 10.1186/s12859-020-03845-3

**Published:** 2020-12-14

**Authors:** Carla Rezende Barbosa Bonin, Guilherme Côrtes Fernandes, Reinaldo de Menezes Martins, Luiz Antonio Bastos Camacho, Andréa Teixeira-Carvalho, Licia Maria Henrique da Mota, Sheila Maria Barbosa de Lima, Ana Carolina Campi-Azevedo, Olindo Assis Martins-Filho, Rodrigo Weber dos Santos, Marcelo Lobosco, Collaborative Group for Studies of Yellow Fever Vaccine

**Affiliations:** 1 Institute of Education, Science and Technology of Southeast of Minas Gerais - Cataguases Advanced Campus, Chácara Granjaria, s/n - Granjaria, 36773-563 Cataguases, Brazil; 2 Medical School, Presidente Antônio Carlos University, Juiz de Fora, Brazil; 3 Bio-Manguinhos Oswaldo Cruz Foundation (FIOCRUZ), Rio de Janeiro, Brazil; 4 Sergio Arouca National School of Public Health (ENSP), Oswaldo Cruz Foundation (FIOCRUZ), Rio de Janeiro, Brazil; 5 Rheumatology Department, University of Brasilia (UnB), Brasilia, Brazil; 6 René Rachou Research Center, Oswaldo Cruz Foundation (FIOCRUZ)/Minas, Belo Horizonte, Brazil; 7 Graduate Program in Computational Modeling, Federal University of Juiz de Fora (UFJF), Juiz de Fora, Brazil

**Keywords:** Vaccine, Yellow fever, Mathematical modeling, Computational modeling, Immune system, Ordinary differential equations

## Abstract

**Background:**

An effective yellow fever (YF) vaccine has been available since 1937. Nevertheless, questions regarding its use remain poorly understood, such as the ideal dose to confer immunity against the disease, the need for a booster dose, the optimal immunisation schedule for immunocompetent, immunosuppressed, and pediatric populations, among other issues. This work aims to demonstrate that computational tools can be used to simulate different scenarios regarding YF vaccination and the immune response of individuals to this vaccine, thus assisting the response of some of these open questions.

**Results:**

This work presents the computational results obtained by a mathematical model of the human immune response to vaccination against YF. Five scenarios were simulated: primovaccination in adults and children, booster dose in adult individuals, vaccination of individuals with autoimmune diseases under immunomodulatory therapy, and the immune response to different vaccine doses. Where data were available, the model was able to quantitatively replicate the levels of antibodies obtained experimentally. In addition, for those scenarios where data were not available, it was possible to qualitatively reproduce the immune response behaviours described in the literature.

**Conclusions:**

Our simulations show that the minimum dose to confer immunity against YF is half of the reference dose. The results also suggest that immunological immaturity in children limits the induction and persistence of long-lived plasma cells are related to the antibody decay observed experimentally. Finally, the decay observed in the antibody level after ten years suggests that a booster dose is necessary to keep immunity against YF.

## Background

At the time this paper was written, a significant global outbreak of COVID-19 was in course. This pandemic clearly illustrates the need for new tools to assist the fast development of vaccines against emerging or unknown diseases. Even vaccines developed decades ago, such as the yellow fever vaccine (YFV), could benefit from new tools.

Although YFV is considered safe, there are rare but serious adverse effects that need to be reassessed, such as viscerotropic and neurotropic events [1]. There are also questions regarding the safety of vaccinating specific populations such as the elderly, people living with Human Immunodeficiency Virus (HIV)/AIDS, and other immunocompromised populations. Studies suggest that the immunological immaturity of infants and young children limits the induction/persistence of long-lived plasma cells [2] and, for this reason, a booster dose is needed. The same occurs with the elderly due to immunosenescence.^1^

In the vaccinology field, computer tools have been used to assist the vaccine development process [4–13]. Several computational modelling techniques can be used to achieve this objective [14]. Most of them focus on non-clinical trials. In previous work, we proposed a novel application of computer tools to vaccinology in the clinical development stage [15]. With mathematical and computational models, it is possible to evaluate in silico different scenarios related to vaccination and answer important questions which remain open, such as the minimal dose that confers immunity and immunity duration. The idea of using computer tools during the clinical development stage was then applied to model the immune response to the YFV [1, 16]. Results showed that mathematical models could capture the immune response to the YFV, and in subsequent work the model was validated quantitatively [17]

This work presents new numerical experiments showing that our model can reproduce experimental data from scenarios such as booster dose, immune response in individuals under immunomodulatory therapy, and primovaccination in children. We also discuss, in more detail, simulations for primovaccination in adults and dose-response, extending the initial results obtained in a previous work [17].

Another work in the literature also uses an ODE-based approach to model the human immune response to vaccination against both YF and smallpox [18] using distinct data and equations sets, one for each disease. The authors aimed to primarily evaluate the dynamics of CD8+ T cells, while our work evaluates the immune response as a whole. The model proposed here differs from that presented by Le et al. [18], since it considers important populations at each stage of the immune response to YF vaccination, from virus inoculation to antigen presentation and consequent activation of lymphocytes, generation of antibodies, and memory cells. Furthermore, our validated model is a great tool to assist specialists in answering some open questions regarding YFV, which were not taken into account by Le et al. [18].

## Results

This section presents the predictions of the mathematical model presented in “Methods” section, comparing them with experimental data from several studies conducted by the Immunobiological Technology Institute (Bio-Manguinhos)/Oswaldo Cruz Foundation (Bio-Manguinhos/FIOCRUZ), René Rachou Research Center/Oswaldo Cruz Foundation (FIOCRUZ/Minas) and University of Brasilia (UnB) on human YFV [3, 19–25], such as viremia and antibody titers,^2^ for distinct scenarios. For all experimental data, we present antibody interquartile range, lower limit, and upper limit. In order to facilitate comparison with numerical results, we also present the geometric mean of the experimental antibody titers (GMT—Geometric Mean Titers).

The first scenario simulates an adult individual being vaccinated for the first time with the full dose of the vaccine against YF. The second scenario represents the revaccination of adult individuals. There are situations in which some individuals’ immune response differs from the response usually obtained by vaccination in immunocompetent adults. This is the case of children and individuals with autoimmune diseases, respectively, the third and fourth scenarios. Finally, the fifth scenario evaluates the use of different doses of the vaccine against YF, all below the full dose.

Numerical results are presented and compared to experimental data [3, 19–25]. More specifically, experimental results from primary vaccination in adults, booster dose in adults, primovaccination in children and individuals using immunomodulatory therapy, and dose-response studies are used to qualitatively and quantitatively validate the numerical results obtained by the mathematical model. A quantitative comparison was performed when experimental and numerical results were in the same unit. However, in some scenarios, the results generated by the model and the experimental data are in different units, mIU/mL, and reciprocal dilution, respectively. This is due to the experimental method used. Neutralising antibody levels in serum was measured by the Plaque Reduction Neutralisation Test (PRNT), either in reciprocal dilution or in International Units. If the standard serum for quantification in International Units is available, this unit’s values are also obtained. What often occurs is the lack of this serum and, consequently, the lack of values in the mIU/mL unit, which precludes a quantitative comparison. For these cases, graphics are constructed with two Y-axes, each representing a unit. The experimental data in reciprocal dilution will be represented by the Y-axis on the left, while the results obtained by the model simulation, in mIU/mL, will be represented by the Y-axis on the right. This allows for a qualitative assessment of the model’s results, by comparing the trends it predicts with those observed experimentally.

### First scenario: primovaccination in adults

The first scenario was used to calibrate the model. In other words, its parameters and initial conditions were chosen to reproduce the experimental results of an individual vaccinated for the first time against YF using the full dose of the vaccine developed by Bio-Manguinhos/Fiocruz (17DD-YFV). After the model was calibrated, most of the parameters and initial conditions values found were kept for the experiments presented in the next sections.

After vaccination, the antibody levels of the subjects who participated in the experiment were measured at different times. These samples were grouped in the following way:

- NV (day 0): Naïve (NV), immediately before vaccination;
- PV (30–45 days): primo-vaccinated (PV), 30–45 days after vaccination;
- PV (1–5 years): 1–5 years after vaccination;
- PV (> 5–9 years): 5–9 years after vaccination;
- PV (10 years): 10 years or more after vaccination.

These groups, in general, will also be used for other studies that will be described in the following sections.

**Fig. 1:**
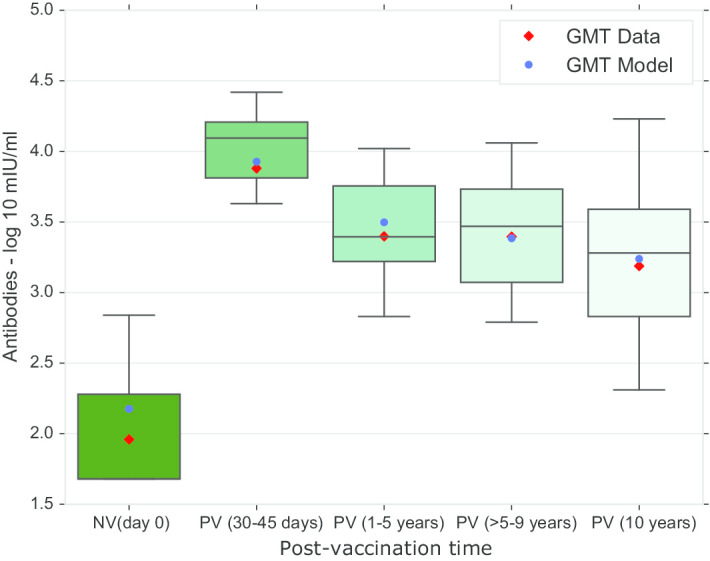
First scenario: primovaccination in adults. interquartile range (rectangles), median (black line), lower limit and upper limit (black stems), and geometric means (observed and estimated by model) of antibody titers for YF according to post-vaccination time. “GMT Data” (  ) refers to the geometric mean of the experimental data and “GMT Model” (  ) refers to the geometric mean of the numerical results

Figure 1 shows the comparison between the levels of antibodies obtained experimentally and numerically, after calibration. These are cross-sectional data so that different individuals will be represented in the categories of post-vaccination time described above and the same categories are used to present numerical results. A pattern of marked increase in antibody levels 30–45 days after vaccination and a reduction, which was more pronounced after 1–5 years but was sustained for 10 years after vaccination.

The model errors were computed for each post-vaccination intervals and the results obtained are shown in Table 1. The model errors were small, an evidence that the model is likely suitable, and has been successfully calibrated.

**Table 1:** Model error for each post-vaccination time interval

Post-vaccination time	Model error (%)
30–45 days	1.2
1–5 years	2.8
> 5–9 years	0.3
10 years	1.6

**Fig. 2:**
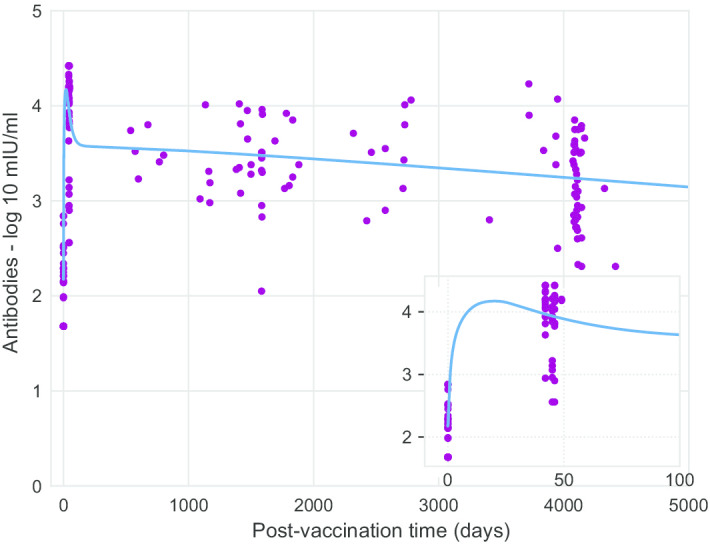
First scenario: primovaccination in adults. comparison between antibody curve (  ) generated by the model for 5000 days of simulation and experimental data (  ) obtained from primo-vaccinated individuals in the same period. The zoom in the figure shows in more details experimental and numerical results for the first 100 days after vaccination

Figure 2 presents experimental data and numerical results for the entire time simulated, which was 5000 days. Although experimental data for this scenario were used to adjust the model, as one can observe, experimental data are restricted to some days. Due to the total simulation time, it is not possible, in main graph, to observe the model results and experimental data for the two initial groups, NV and PV (30–45 days). To facilitate the visualisation of the curve in the first days of simulation, a secondary graphic is presented in the same figure, which presents experimental data only for the first 100 days after vaccination, as well as the numerical results.

It is possible to notice in Fig. 2 that, between days 1 and 41, no experimental data were obtained. Thus, it was not possible to make the adjustment or even evaluate the quality of the curve generated by the model in this interval.

**Fig. 3:**
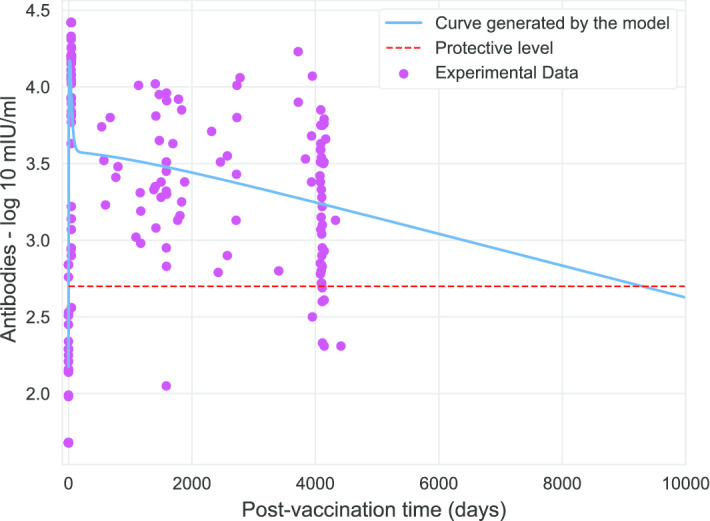
First scenario: primovaccination in adults. Antibody curve (  ) generated by the model for 10,000 days of simulation and experimental data (  ) obtained from primo-vaccinated individuals in the same period. The red dashed line (  ) presents the protective level

A booster dose is required if the antibody level is below the the seropositivity threshold. Figure 3 presents the simulation for 10,000 days after the first vaccination. As one can observe, the curve generated by the model for a single dose suggests that the amount of antibodies is below the protective level about 10,000 days after vaccination, thus indicating the need of administration of a booster dose.

### Second scenario: booster dose in adults

In addition to assessing the immune response to the first dose of the YFV, the Fiocruz research group also collected experimental data from revaccinated (RV) individuals. In this study, the antibody levels of the subjects who participated in the experiment were measured at different times, and these samples were grouped in the following way:

- PV (> 5–9 years): 5–9 years after first vaccination;
- PV (10 years): 10 years or more after first vaccination;
- RV (30–45 days): 30–45 days after booster dose;
- RV (1–5 years): 1–5 years after booster dose;
- RV (> 5–9 years): 5–9 years after booster dose;
- RV (10 years): 10 years or more after booster dose.

Data obtained for this scenario were used to validate the model, without changing or adjusting the parameters and initial values found during calibration. For this purpose, the following method was adopted. Initially, a simulation of a primo-vaccinated individual was performed. After simulating the equivalent of 5500 days since the application of the vaccine, the simulation was paused, the current values for all populations of the model were saved and only the value associated to the virus population was modified, from its current value, zero, to the adjusted full vaccine dose. The simulation was then resumed, 4500 additional days were simulated to reach 10,000 days.

These specific numbers of days after vaccination, 5500 and 10,000, were chosen based on experimental data available. The PV group (10 years), that is, adult individuals 10 years after the first vaccine dose, had samples collected up to 5475 days (15 years) after vaccination. For this reason, the booster dose was simulated in the model 5500 days after the first dose. Likewise, in the RV group (10 years), individuals 10 years after the booster dose, had samples collected up to 3650 days after the second dose, and consequently up to 9125 days after the first dose. For this reason, the simulation was for 10,000 days after application of the first dose.

**Fig. 4:**
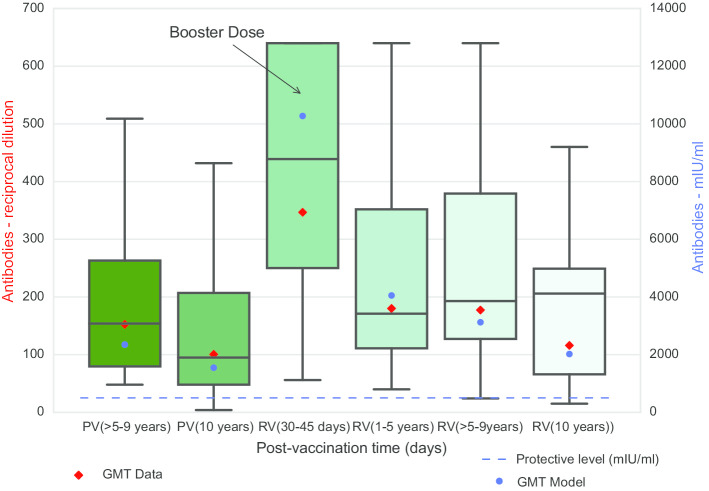
Second scenario: booster dose in adults. Interquartile range (rectangles), median (black line), lower limit and upper limit (black stems) and geometric means (observed and estimated by model) of antibody titers for YF according to post-vaccination time, first dose and booster dose. “GMT Data” (  ) refers to the geometric mean of the experimental data and “GMT Model” (  ) refers to the geometric mean of the numerical results. Experimental data expressed in reciprocal dilution, and numerical ones in PRNT mIU/mL. The blue dashed line (  ) presents the protective level expressed in mIU/mL

Figure 4 presents experimental data and numerical results for the booster dose scenario. Units for model predictions (  ) and the experimental data differ, as the latter was only available in terms of reciprocal dilution.

### Third scenario: primovaccination in children

As mentioned in “Background” section, immune response in child is less pronounced than in adults. Some hypotheses explain these differences: immunological immaturity limits the induction and persistence of long-lived plasma cells [27]. Long-lived plasma cells are largely responsible for long-term secretion of antibodies [28].

In this scenario, these two possibilities (limitation of induction and persistence) were evaluated numerically. For this purpose, changes were made only to the values of parameters related to these hypotheses, without any further modification, except for the weight of the individual being simulated and the initial condition for the antibody population. Table 10 shows the weight, and percentage of fluids in the body that was used as a basis for calculating the initial condition of the virus that would be used in simulations of adults and children, as well as the initial amount of antibodies used for each population.

The hypothesis that immunological immaturity limits the persistence of long-lived plasma cells was evaluated increasing the natural death rate for this type of cells (represented by parameter $\delta _{pl}$). However the simulations showed that changes in this value had no significant effect on the antibodies curve and, for this reason, this result was omitted. The hypothesis that the immunological immaturity limits the induction of long-lived plasma cells was tested reducing the rate of differentiation of B cells into long-lived plasma cells ($\beta _{pl}$). There was a noticeable reduction in the lifelong memory by changing only this parameter. The simultaneous alteration of $\beta _{pl}$ and $\delta _{pl}$ was also evaluated. Although the change in $\delta _{pl}$ alone did not produce a significant reduction in antibodies, when combined with changes in $\beta _{pl}$ value, the results produced the best fit to reproduce experimental data, which are described in this section.

**Fig. 5:**
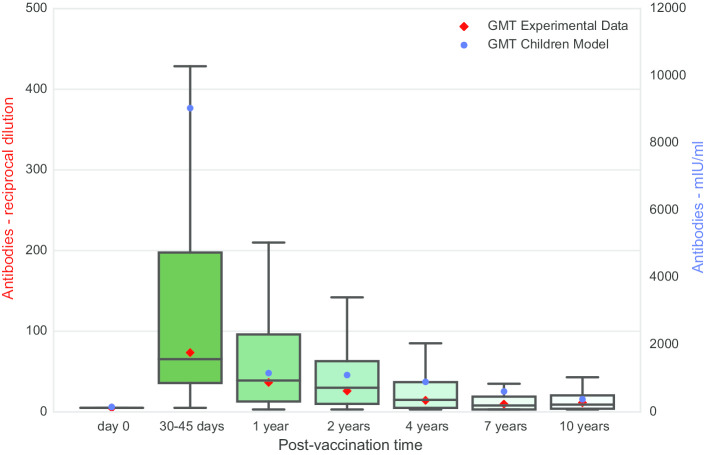
Third scenario: primovaccination in children. Interquartile range (rectangles), median (black line), lower limit and upper limit (black stems) and geometric means (observed and estimated by the model) of antibody titers for YF after vaccination in children. “GMT Experimental Data” (  ) refers to the geometric mean of children’s vaccination data and “GMT Model Children” (  ) to the geometric mean of numerical results after parameters has been adjusted to represent the immune response of children. Experimental data expressed in reciprocal dilution, and numerical ones in PRNT mIU/mL

A range of values were tested for $\beta _{pl}$ and $\delta _{pl}$. The simulation using a reduction of approximately 70% of the value associated to the parameter $\beta _{pl}$ and an increase of 100% of the value associated to the parameter $\delta _{pl}$ used for adults produced the best fit to reproduce qualitatively the immune response of children. The values of $\beta _{pl}$ in the model were $1.68\times 10^{-6}$ and $5.61\times 10^{-6}$ to simulate children and adults, respectively. The values of $\delta _{pl}$ in the model were $2.4\times 10^{-4}$ and $4.8\times 10^{-4}$ to simulate children and adults, respectively. Figure 5 shows that numerical results were able to reproduce the same behaviour observed in experimental data: an initial rapid increase in the amount of antibodies is followed by a decrease over the course of time. It should also be noted that these are cross-sectional data, so that antibody levels in post-vaccination times are from different children.

**Fig. 6:**
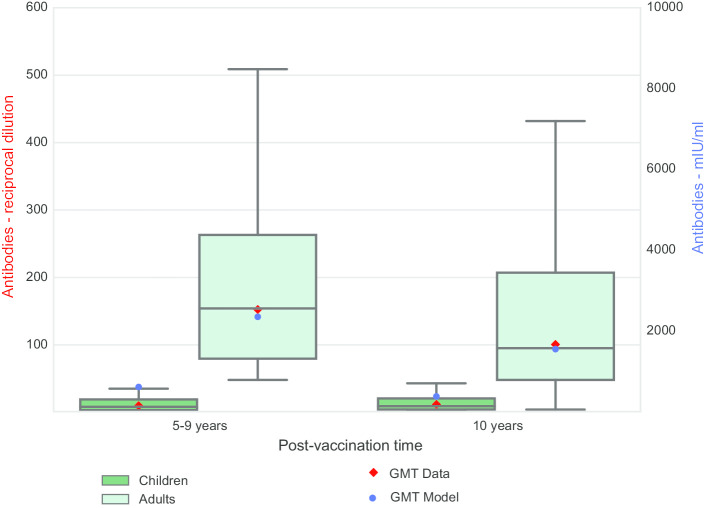
Third scenario: primovaccination in children. Antibody levels generated by the model (for adults and children) and experimental data for adults and children. “GMT Data” (  ) refers to the geometric mean of data (for adults and children) and “GMT Model” (  ) to the geometric mean of numerical results (for adults and children). Experimental data expressed in reciprocal dilution, and numerical ones in PRNT mIU/mL

To facilitate the comparison between the immune response of adults and children when vaccinated against YF, experimental data and numerical results for these two groups are shown in Fig. 6. For adults and children, the experimental data in reciprocal dilution (Y-axis on the left) allow direct comparison. Conversely, results from the numerical experiment in mIU/mL (Y-axis on the right), allow comparisons of patterns only.

### Fourth scenario: immune response in individuals using immunomodulatory therapy

A study found in the literature [25] reports differences in the immune response to the YFV in groups of individuals using different types of immunomodulatory therapies. The therapies covered in the study are divided into two main groups, those that use only synthetic DMARDs (disease-modifying antirheumatic drugs) and those that use a combination of biological and synthetic drugs. According to the study [25], DMARDs have the ability to modify or affect the pre-existing protective immunity induced by the vaccine, including the function of memory T and B cells and, as a consequence, the neutralising antibody levels specific to YF. The biggest difference was found when comparing the control group, that is, individuals without any autoimmune disease, with the group using combination therapy.

The hypotheses found in the literature to explain how DMARDs affects the pre-existing protective immunity induced by the vaccine [25] were evaluated using the model. Again, changes were made only in the parameters related to the hypotheses, keeping the other values found during calibration. Simulations of individuals in two conditions, control and under the use of combination therapy, were carried out. For simulating individuals under use of combination therapy, changes were tested in the values of all parameters of the equation that describes the dynamics of B cells, as well as in their initial conditions. The following alterations were able to reproduce the antibody levels of individuals using combination immunomodulatory therapy:

- $50\%$ reduction in $\alpha _b$ parameter (B cell homeostasis rate);
- $25\%$ reduction in parameter $\beta _{pl}$ (B cell differentiation rate in long-lived plasma cells);
- $25\%$ reduction in B cell initial condition.

These three alterations, reduction in $\alpha _b$, reduction in $\beta _{pl}$, and reduction in B cell initial condition, produced very similar results: all of them reproduced the immune response of an individual with autoimmune disease using combination immunomodulatory therapy. For this reason, only one of the results is presented in this section, the one which reduces the initial condition of B cells by 25%. The reduction percentages were chosen after carrying out several tests with distinct values for the parameters and initial condition of B cells. The values that produced the best adjustments in the levels of antibodies generated by the model to the experimental data obtained for the individuals in use of combination immunomodulatory therapy were chosen and are shown in Table 2.

**Table 2:** Values of parameters* α*_*b*_,* β*_*pl*_ and *B*0 used in the model to simulate control subjects and those using combination immunomodulatory therapy

Parameter	cs $+$ bDMARD value	Control value
$\alpha _b$	3.0	6.0
$\beta _{pl}$	$4.208\times 10^{-6}$	$5.61\times 10^{-6}$
*B*0	$1.875\times 10^{5}$	$2.5\times 10^{5}$

Each line represents a distinct and independent adjust, *i.e.*, the modification of a single parameter at a time is able to approach experimental data

**Fig. 7:**
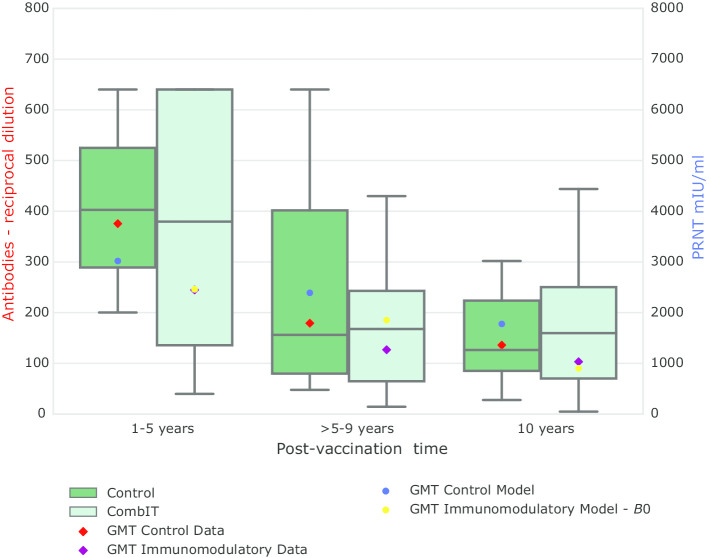
Fourth scenario: immune response in individuals using immunomodulatory therapy. Interquartile range (rectangles), median (black line), lower limit and upper limit (black stems) and geometric means (observed and estimated by the model) of YF antibody titers according to post-vaccination time, for control individuals and those under use of combination immunomodulatory therapy. “GMT Control Data” (  ) refers to the geometric mean of experimental data obtained from control individuals and “GMT Control Model” (  ) to the geometric mean of numerical results obtained when using the original parameters values. “GMT Immunomodulatory Data” (  ) refers to the geometric mean of experimental data obtained from individuals using the therapy and “GMT Immunomodulatory Model” (  ) to the geometric mean of the numerical results obtained when using the adjusted parameters. Experimental data expressed in reciprocal dilution, and numerical ones in PRNT mIU/mL

Figure 7 presents experimental and numerical data for control individuals and those using immunomodulatory therapy. In this figure, the numerical results modify only the value associated to the initial condition of B cells (*B*0).

### Fifth scenario: dose-response

The literature [3] reported that doses from 27,476 IU to 587 IU of the YFV induced seroconversion rates and similar GMT in the participants of the experiment. Based on that study, we simulated the immune response after the administration of different doses of the YFV. This was done changing the values used as the initial virus condition in the model to be the same described in the literature [3]. These values adopted as initial condition were computed considering the dilution of the vaccine in the body, as well as the conversion of the units, as presented in “Experimental data” section. The values of all other parameters were kept the same.

**Fig. 8:**
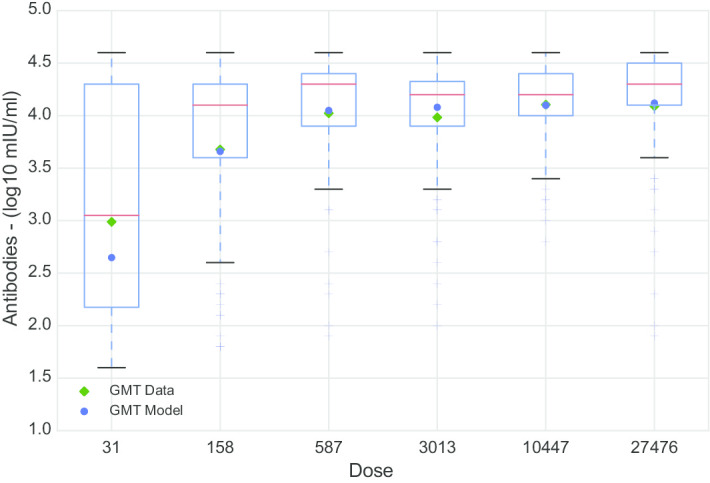
Fifth scenario: dose-response. Interquartile range (rectangles), median (red line), lower limit and upper limit (black stems) and GMT (observed and estimated by model) for different doses of vaccine (31 IU, 158 IU, 587 IU, 3013 IU, 10,447 IU and 27,476 IU) within 30–45 days after vaccination. “GMT Data” (  ) refers to the geometric mean of the experimental data and “GMT Model” (  ) refers to the geometric mean of the numerical results, both obtained for each dose

Mean antibody titers 30–45 days after vaccination generated by model simulation approximated the actual data in the dose-response study, which also used International Units (Fig. 8). The data showed that antibody levels increased with vaccine doses up to 587 mIU, above which no further increase in antibody levels was achieved.

**Fig. 9:**
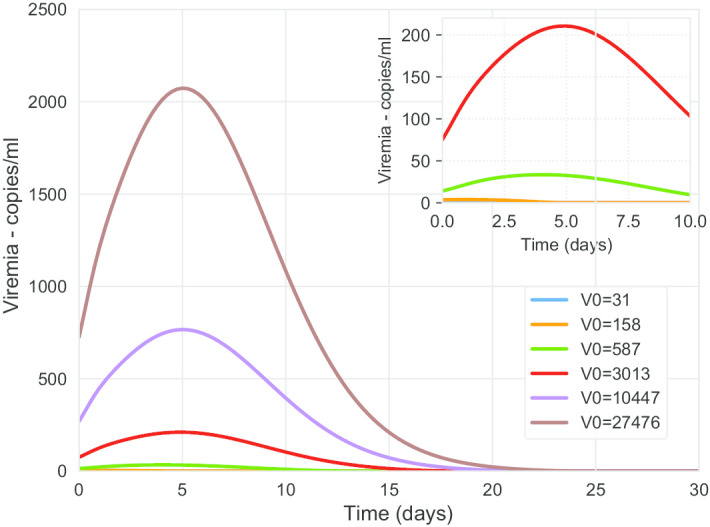
Fifth scenario: dose-response. Simulation of antibody levels curves until 1000 days of vaccination, considering distinct vaccine doses (31 IU, 158 IU, 587 IU, 3,013 IU, 10,447 IU and 27,476 IU)

Antibody levels generated by the model (Fig. 9) showed a pattern of marked increase with a peak within 20 days of vaccination, somewhat earlier and much lower for vaccine doses 31 mIU and 158 mIU. According to the model, vaccine doses 587 mIU and above induced and sustained similar antibody levels for 1000 days. The main graphic presents the antibody levels curves until 1000 days of vaccination. To better observe the curve behaviour in the first days after vaccination, a secondary graphic on the upper right presents the same result for the first 100 days after vaccination.

**Fig. 10:**
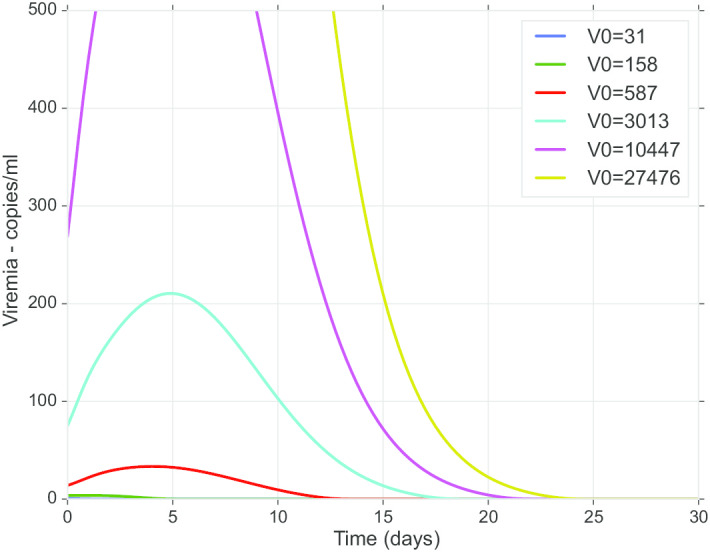
Fifth scenario: dose-response. Simulation in viremia curves considering distinct vaccine doses (31 IU, 158 IU, 587 IU, 3,013 IU, 10,447 IU and 27,476 IU)

Figure 10 presents the numerical results for viremia curves, considering distinct vaccine doses. The main graphic does not allow a detailed observation of the curves for the smaller doses and, for this reason, a secondary graphic on the upper right presents a zoom in this figure, allowing one to observe that viremia for some of smaller doses is not equal to zero.

## Discussion

The immune response to vaccination was successfully modelled in several of its relevant components presented in different scenarios. In general, the immune response described by the model provided a reasonable approximation of empirical data showing that it was built on sound mathematical relations of the key parameters.

The first scenario, primovacciation in adults, was used to adjust model parameters and initial conditions. As one could expect, the observed error presented in Table 1 was very low, below 3%. For the fifth scenario, the dose-response experiment, except for the lowest dose whose error was about 13%, for all other doses the errors were bellow 2.5%, as Table 3 reveals. This result showed that the model, that was adjusted using only data from individuals vaccinated with the full dose, was able to satisfactorily reproduce the immune response obtained with vaccination using doses lower than the full one. For all other simulated scenarios, it was not possible to make a similar quantitative analysis either because data were not available, or because units were different. Experimental data available for antibody levels use reciprocal dilution as unit, while the model uses mIU/mL, and one unit cannot be converted into the other one with available data. Thus, it is not possible to say, for example, that an increase of 50% in the level of antibodies expressed in reciprocal dilution means an increase of the same 50% expressed in mIU/mL.

**Table 3:** Model error for each dose between 30 and 45 days post-vaccination

Dose (IU)	Model error (%)
31	12.9
158	0.5
587	0.7
3013	2.4
10,447	0.1
27,476	0.7

A similar pattern in experimental data and model outputs was observed in Fig. 4, in “Second scenario: booster dose in adults” section. Reduced antibody levels in individuals vaccinated 5–9 and 10 years before, were followed by a pronounced rise after a booster dose and a marked reduction after 1–5 years. Antibody levels 10 years after revaccination were almost as low as those before the booster dose.

Despite the difference in the units adopted, it was possible to notice in Fig. 5, in “Third scenario: primovaccination in children” section, that the numerical results were able to reproduce the same behaviour observed in experimental data: an initial rapid increase in the amount of antibodies is followed by a decrease over time.

As presented in Fig. 6, also in “Third scenario: primovaccination in children” section, experimental data showed that there is an evident difference in the levels of antibodies produced by adults and children. It should be noted that the model was adjusted using data from adults expressed in mIU/mL, and therefore it is not the same unit used in experimental data, which are expressed in reciprocal dilution. Still, in a qualitative way, the numerical results were able to capture this behaviour: a lower level of antibodies in children than in adults.

Three reductions in constant/initial condition values ($\alpha _b$, $\beta _{pl}$, and B cell initial condition) numerically evaluated in this work could explain the immune response in individuals using immunomodulatory therapy. These results could change if other aspects of the way DMARDs work in the body, and its mechanisms of interaction with each type of cell, were also considered. Some mechanisms involve the production and/or inhibition of cytokines that were not yet considered in the model.

The model was able to reproduce distinct scenarios related to the immune response to vaccination against YF. For this reason, we decided to use it to obtain some clues about the questions that remain unanswered or poorly understood about the vaccine. The first clue is that, among all evaluated doses, the lowest dose capable of conferring immunity is half of the minimum recommended by the WHO, as the numerical experiments in “Fifth scenario: dose-response” section show. The results presented in Fig. 9 show that the antibody curve is almost the same for all doses above 587 IU; these results are similar to those presented in the literature [3].

The second clue is related to the hypothesis that immunological immaturity in children limits the induction and persistence of long-lived plasma cells. Numerical results confirmed that both are responsible for the differences observed in experimental results of adults and children, and that persistence of long-lived plasma had no significant effect on the antibody curves alone.

The third clue is related to the need of booster dose. A single dose apparently (as suggested by the results in Fig. 3) did not provide long lasting protection. The decay rate in the antibody level suggests that the booster dose is needed to maintain protection. In fact, about 10,000 days after vaccination, the level of antibodies in an adult is below the protective level if a single dose is given. If a booster dose is given, the protection level is improved, as depicted in Fig. 4. Moreover, the single dose is usually given to infants or children, which induces a lower amount of long-lived plasma cells than adults, which reinforces the need of booster doses throughout life.

Some considerations and limitations of the model used in this study should be highlighted. The model was adjusted to reflect the geometric mean of the experiments. In this sense, conclusions reflect the typical immune response from the average individual described in Tables 10 and  11. Some individuals with distinct characteristics, such as the immunological immaturity of children or a compromised immune system due to some disease should, for example, receive a booster dose of the YFV in a shorter period of time. Furthermore, the extrapolation done to predict the antibody level after 10,000 days may suffer from the classical overfitting problem, where the model can replicate the data it is adjusted to but fails on any attempt of extrapolation or forecasting. Finally, other aspects that may influence the minimal dose to confer immunity against YF were not taken into account, such as problems with virus die-off during transport.

## Conclusions

This work presented the quantitative and qualitative validation of a mathematical-computational model to represent the immune response to the YFV using five distinct scenarios. The first one simulates the immune response to the administration of the full dose of the 17DD-YFV for the first time. The second one simulates the immune response to distinct doses of vaccine. The third scenario simulates the administration of a booster dose ten years after the first dose. The fourth simulates the vaccination in individuals under immunomodulatory therapy. Finally, the last one simulates the primary vaccination in children. The numerical results were collected and compared to experimental data. Some results could be compared directly, and errors below 10% were observed. For other results that could not be compared directly, because distinct units were used, it was observed that the numerical results obtained by the computational model satisfactorily reproduced the behaviour observed in experimental data.

The numerical experiments show that among all vaccine doses evaluated, the lowest one capable of conferring immunity against YF is about half of the reference dose, 587 UI. The results also suggest that the hypothesis that the immunological immaturity in children limits long-lived plasma cells’ persistence is not related to the antibody decay observed experimentally. The numerical experiments show that this phenomenon is due to the lower induction of long-lived plasma cells. Finally, the antibody level’s decay within the ten years following vaccination suggests that a booster dose is necessary to keep immunity against YF.

Although the model presented in this work focuses on the YFV, it could be used to gain new insights in the immune response to vaccine canditates, such as those for COVID-19.

We also plan, as future work, to refine the model to guide future empirical studies: (1) to determine the optimal number of doses to ensure protection against YF; (2) to determine the duration of immunity with two vaccine doses in infants; (3) to determine the interval between these two doses given to infants to maximise the duration of immunity, and (4) conduct a dose-response study in infants.

## Methods

### Mathematical model

The model used in this work consists of a system of ordinary differential equations (ODEs), which were originally proposed in a previous work [1, 17], and reproduced here. These equations represent the main populations involved in the immune response to the vaccination, as well as the virus itself. They are yellow fever vaccine virus, APCs (Antigen-presenting cells), CD4$+$ T cells, CD8$+$ T cells, short and long-lived plasma cells, B cells, memory B cells, and antibodies. The initial conditions and acronyms of these populations, as well as the parameters and their meanings, are presented in Tables 11 and 12, respectively, which are presented in “Appendix”.

Equation (1) represents the vaccine virus (*V*):

1 $$\begin{aligned} \frac{d}{dt}V= \pi _v V - \frac{c_{v1} V}{c_{v2} + V} - k_{v1} V A - k_{v2} V T_{ke}. \end{aligned}$$

The virus can not proliferate by itself. It needs to infect a cell and use it as a factory for new viruses. This mechanism is implicitly considered in the term $\pi _v V$, which represents the multiplication of the virus in the body, with a production rate $\pi _v$. The term $\frac{c_{v1} V}{c_{v2} + V}$ denotes a non-specific viral clearance made by the innate immune system. This function models growth combined with a saturation effect [29]. The term $k_ {v1} V A$ denotes specific viral clearance due to antibody signalling, where $k_ {v1}$ is the clearance rate. The term $k_ {v2} V T_{ke}$ denotes specific viral clearance due to the induction of apoptosis of cells infected by the YF virus, where $k_ {v2}$ is the clearance rate.

APCs are all cells that display antigens complexes on their surfaces, such as dendritic cells and macrophages. Two stages of APCs were considered: immature and mature. The first stage, immature APCs ($A_{p}$), is described by Eq. (2):

2 $$\begin{aligned} \frac{d}{dt}A_{p}= \alpha _{ap} (A_{p0} - A_p) - \beta _{ap} A_p \frac{c_{ap1} V}{c_{ap2} + V}. \end{aligned}$$

The term $\alpha _{ap} (A_{p0} - A_p)$ describes the homeostasis of APCs, where $\alpha _{ap}$ is the homeostasis rate. The term $\beta _{ap} A_p \frac{c_{ap1} V}{c_{ap2} + V}$ denotes the conversion of immature APCs into mature ones. So the same term appears in Eq. (3) with positive sign.

The Eq. (3) represents the mature APCs ($A_{pm}$):

3 $$\begin{aligned} \frac{d}{dt}A_{pm}= \beta _{ap} A_p \frac{c_{ap1} V}{c_{ap2} + V} - \delta _{apm}A_{pm}. \end{aligned}$$

The first term, as just explained, denotes the dynamics of APCs maturation. The second term, $\delta _{apm}A_{pm}$, means the natural decay of the mature APCs, where $\delta _{apm}$ is the decay rate.

Equation (4) represents the population of naïve CD4+ T cells ($T_{hn}$):

4 $$\begin{aligned} \frac{d}{dt}T_{hn}= \alpha _{th} (T_{hn0} - T_{hn}) - \beta _{th} A_{pm} T_{hn}. \end{aligned}$$

The term $\alpha _{th} (T_{hn0} - T_{hn})$ represents the homeostasis of CD4+ T cells, where $\alpha _{th}$ is the homeostasis rate. The term $\beta _{th} A_{pm} T_{hn}$ denotes the activation of naïve CD4+ T cells, where $\beta _{th}$ is the activation rate.

Equation (5) represents the effector CD4+ T cell population ($T_{he}$):

5 $$\begin{aligned} \frac{d}{dt}T_{he}= \beta _{th} A_{pm} T_{hn} + \pi _{th} A_{pm} T_{he} - \delta _{th} T_{he}. \end{aligned}$$

The term $\pi _{th} A_{pm} T_{he}$ represents the proliferation of effector CD4+ T cells. The term $\delta _{th} T_{he}$ represents the natural death of these cells, with $\delta _{th}$ representing its decay rate.

The mechanisms used to represent CD4+ T cells were also used to model the dynamics of CD8+ T cells. Equations (6) and (7) represent the population of naïve ($T_{kn}$) and effector ($T_{ke}$) CD8+ T cells:

6 $$\begin{aligned} \frac{d}{dt}T_{kn}=\,\alpha _{tk} (T_{kn0} - T_{kn}) - \beta _{tk} A_{pm} T_{kn}, \text {and} \end{aligned}$$

7 $$\begin{aligned} \frac{d}{dt}T_{ke}= \,\beta _{tk} A_{pm} T_{kn} + \pi _{tk} A_{pm} T_{ke} - \delta _{tk} T_{ke}. \end{aligned}$$

The term $\alpha _{tk} (T_{kn0} - T_{kn})$ represents the homeostasis of CD8+ T cells, where $\alpha _{tk}$ is the homeostasis rate. The term $\beta _{tk} A_{pm} T_{kn}$ denotes the activation of naïve CD8+ T cells, where $\beta _{tk}$ is the activation rate. The term $\pi _{tk} A_{pm} T_{ke}$ represents the proliferation of effector CD8+ T cells, where $\pi _{tk}$ is the activation rate. The term $\delta _{tk} T_{ke}$ represents the natural death of these cells, with $\delta _{tk}$ representing its decay rate.

Equation (8) represents B cells (*B*), both naïve and effector ones. These populations were not considered separately in order to simplify the model.

8 $$\begin{aligned} \begin{aligned} \frac{d}{dt}B&= \alpha _b (B_0 - B) + \pi _{b1} V B + \pi _{b2} T_{he} B- \beta _{ps} A_{pm} B \\&\quad - \beta _{pl} T_{he} B - \beta _{bm} T_{he} B. \end{aligned} \end{aligned}$$

The term $\alpha _b (B_0 - B)$ represents the B cells homeostasis, where $\alpha _b$ is the homeostasis rate. The terms $\pi _{b1} V B$ and $\pi _{b2} T_{he} B$ represent the proliferation of B cells activated by the T-cell independent and T-cell dependent mechanisms, respectively. The terms $\beta _{ps} A_{pm} B$, $\beta _{pl} T_{he} B$ and $\beta _{bm} T_{he} B$ denote the differentiation of active B cells into short-lived plasma cells, long-lived plasma cells and memory B cells, respectively. The activation rates are respectively given by $\beta _{ps}$, $\beta _{pl}$ and $\beta _{bm}$.

Equation (9) represents the short-lived plasma cells ($P_s$):

9 $$\begin{aligned} \frac{d}{dt}P_s= \beta _{ps} A_{pm} B - \delta _{ps} P_s. \end{aligned}$$

The term $\delta _{ps} P_s$ denotes the natural decay of short-lived plasma cells, where $\delta _{ps}$ is the decay rate.

Equation (10) represents the long-lived plasma cells ($P_l$):

10 $$\begin{aligned} \frac{d}{dt}P_l= \beta _{pl} T_{he} B - \delta _{pl} P_l + \gamma _{bm} B_m. \end{aligned}$$

The term $\delta _{pl} P_l$ denotes the natural decay of long-lived plasma cells, with $\delta _{pl}$ representing the decay rate. The term $\gamma _{bm} B_m$ represents the resupply of these cells by memory B cells, where $\gamma _{bm}$ is the production rate.

Eq. (11) corresponds to memory B cells ($B_m$):

11 $$\begin{aligned} \frac{d}{dt}B_m= \beta _{bm} T_{he} B + \pi _{bm1} B_m\left( 1 - \frac{B_m}{\pi _{bm2}}\right) - \gamma _{bm} B_m. \end{aligned}$$

The term $\pi _{bm1} B_m\left( 1 - \frac{B_m}{\pi _{bm2}}\right)$ represents the logistic growth of memory B cells, *i.e.*, there is a limit to this growth. $\pi _{bm1}$ represents the growth rate, and $\pi _{bm2}$ limits the growth.

Finally, Eq. (12) represents the antibodies:

12 $$\begin{aligned} \frac{d}{dt}A= \pi _{ps} P_s + \pi _{pl}P_l - \delta _a A. \end{aligned}$$

The terms $\pi _{ps} P_s$ and $\pi _{pl}P_l$ are the production of the antibodies by the short-lived and long-lived plasma cells, respectively. The production rates are given by $\pi _{ps}$ and $\pi _{pl}$, respectively. The term $\delta _a A$ denotes the natural decay of these cells, where $\delta _a$ is the decay rate.

### Computational model

The model was implemented in the Python programming language. Numerical solution of the system of ODEs performed by the odeint function, a member of the integrate package in the scipy library [30]. This function uses the characteristics of the ODE system to select the numerical method used, with adaptivity in both timestep and convergence order.

The experiments were performed using Python version 3.7.5 using the Spyder integrated development environment (IDE). The execution environment was composed of an Intel Core i5 1.6 GHz processor, with 8 GB of RAM. The system runs macOS Mojave version 10.14.6.

### Experimental data

The first set of experimental data used was the one that presents markers of the immunological response to the vaccine against YF in adults who were primed and revaccinated. The Tables 4 and  5 present a summary of data that were used for the primed individuals [31] and Table 6 for revaccinated individuals [24]. The antibody data presented in the tables represent the geometric mean of the antibody titers (GMT - Geometric Mean Titers) of all individuals in each group.

**Table 4:** Single dose adults—antibodies (log_10_ mIU/mL)—by time interval

Categorical time	Number of individuals	Average time (days)	GMT ($\text {log}_{10}$ mIU/mL)
NV (0)	46	0	1.96
PV (30–45 days)	46	44 (42–49)	3.88
PV (1–5 years)	36	1367 (537–1833)	3.40
PV (5–9 years)	12	2609 (1882–3406)	3.40
PV (> 10 years)	45	4081 (3721–4414)	3.19

**Table 5:** Single dose adults—antibodies (reciprocal dilution)—by time interval

Categorical time	Number of individuals	Average time (days)	GMT (reciprocal dilution)
PV (5–9 years)	23	2797 (2008–3285)	152
PV (> 10 years)	45	5021 (3650–5475)	100

**Table 6:** Revaccinated—antibodies (reciprocal dilution)—by time interval

Categorical time	Number of individuals	Average time (days)	GMT (reciprocal dilution)
RV (30–45 days)	45	40 (30–69)	347
RV (1–5 years)	47	1017 (365–1825)	180
RV (5–9 years)	34	2287 (2190–2555)	177
RV (> 10 years)	9	3163 (2920–5840)	116

Tables 7 and 8 present a summary of data that were used on vaccination against YF in children [19, 20] and individuals using immunomodulatory therapy [25], respectively. Table 9 summarises data on antibody levels from the study evaluating the dose *versus* response [3, 22, 23].

**Table 7:** Children—antibodies (reciprocal dilution)—by time interval

Categorical time	Number of individuals	Average time (days)	GMT (reciprocal dilution)
NV (0)	50	0	5
PV (30–45 days)	46	39 (30–57)	74
PV (1 year)	113	409 (243–549)	37
PV (2 years)	93	758 (558–909)	26
PV (4 years)	97	1529 (966–2064)	14
PV (7 years)	93	2562 (2379–2982)	10
PV (10 years)	111	3670 (3027–4239)	12

**Table 8:** Use of immunomodulatory therapy—antibodies (reciprocal dilution)—by time interval

Categorical time	Number of individuals	Average time (days)	GMT (reciprocal dilution)
CONT (1–5 years)	4	1553 (1200–1800)	375.55
CONT (> 5–9 years)	26	2745 (1950–3240)	179.30
CONT (10 years)	11	3900 (3450–5520)	136.07
cs + bDMARD (1–5 years)	10	1233 (660–1830)	244.88
cs + bDMARD (> 5–9 years)	24	2686 (2070–3420)	126.81
cs + bDMARD (10 years)	13	5268 (3600–7500)	103.25

**Table 9:** Dose response—antibodies (log_10_ mIU/mL)—by time interval

Dose-IU	Number of individuals	Average time (days)	GMT day 0	GMT PV 30–45 days
31	92	27 (21–34)	2.12	2.99
158	88	27 (20–34)	2.14	3.68
587	92	28 (21–34)	2.16	4.02
3013	100	28 (21–34)	2.14	3.98
10,447	91	28 (21–34)	2.19	4.10
27,476	98	27 (21–34)	2.23	4.09

It is possible to observe in these tables a difference in the unit of the antibody titers (mIU/mL and reciprocal dilution). The test that is normally performed for the quantification of antibodies, PRNT, generates results in reciprocal dilution. When, at the time of testing, the standard serum for quantification in International Units was available, the value in this unit was also obtained. What often occurs was the lack of this serum and consequently the lack of values in mIU/mL.

Thus, in some experiments, the levels of antibodies were expressed in mIU/mL while in others, in the reciprocal dilution. The unit adopted by the model for the concentration of antibodies is mIU/mL and data in that unit were used for a quantitative validation of the model. However, data expressed in reciprocal dilution were also used in the validation of the model, but in a qualitative way.

### Experimental data *versus* numerical results

One of the main changes made in our previous work [1], after access to experimental data, was to adjust the units used in the model. The amount of vaccine virus used as the model’s initial condition was one of these changes. Previously [1], the value 27,476 IU was used, which is the average amount of virus present in the full dose, that is, in 0.5 mL. Variations in the amount of virus across vaccine lots were not considered. But now it is considered that, from the moment the vaccine is injected into an individual, it is diluted in the volume of fluids that the individual has in the body, something around 65% of the body weight (Table 10 shows the weight and percentage of fluids in the body used for adults and children). In addition, in this paper, a comparison between experimental data and the viremia curve generated by the model is done. The unit used in experimental viremia data is copies/mL, that is, number of viral particles per millilitre. To compare numerical and experimental data, both results must be expressed in the same unit. It is then necessary to convert from IU/dose (27,476 IU in 0.5 mL of the dose) to IU/mL of liquid in the body. After that, the value found has to be converted to PFU/mL^3^ using the relationship 1 IU = 1.91 PFU [3]. To convert from PFU/mL to copies/mL, a relationship found in the literature [32], and presented in Eq. (13), was used:

13 $$\begin{aligned} \log _{10} PFU/mL = [0.974 \log _{10} copies/mL] - 2.807. \end{aligned}$$

With respect to other populations, except for antibodies, the values used in our previous work [1] were number of cells found in 1 $\upmu$l, and for this reason, they were multiplied by $10^3$ to be converted to mL. These changes in initial conditions forced us to also readjust the model parameters.

**Table 10:** Values used for simulating adults and children

	Weight (kg)	Liquid in the body %	Antibodies (initial value—mIU/mL)
Adults	85	65	150
Children (9 months)	9	65	0

Finally, it was observed in experimental data that, even before adults were vaccinated, some of them already had antibodies against YF. There are some hypotheses to justify the presence of antibodies prior to vaccination, one of them is the cross protection caused by contact with others *flavivirus*, such as the dengue virus, for example [33]. But perhaps the most likely is a previous non recorded vaccination. Due to this observation, the initial condition of the model that represents the antibody concentration in adults was set to a value similar to that observed experimentally, a value around 150 mIU/mL. For children, this value was defined as zero.

It is necessary to clarify how comparisons between experimental and numerical data were done. For all scenarios (first vaccination in adults, booster dose in adults, primovaccination in children and individuals using immunomodulatory therapy, and dose-response), regardless of the units, data from several individuals exist at different post-vaccination time intervals. For the same day there are one or more individuals and the values found may vary due to differences in immune responses that can be caused by numerous factors such as medication use, genetic inheritance, habits and many others.

Experimental data were already been grouped by post-vaccination time interval and this division was kept. For each group there are individuals spread over the entire time-interval range, which makes it difficult to identify trends in data sets. For this reason, it was decided to present data in a box diagram format (boxplot), thus facilitating a summary view of data for each of the intervals. To each boxplot it has been added the geometric mean of the antibody titers (GMT), defined as the nth root of the product of *n* terms and calculated using the formula presented in Eq. (14):

14 $$\begin{aligned} \left( \prod _{i = 1}^{N} x_i\right) ^{\frac{1}{N}} = \root N \of {x_1 x_2 \ldots x_N}. \end{aligned}$$

To compare experimental and numerical data, not only the geometric mean of the antibody titers were computed for experimental data, but it was also necessary to compute it for the numerical results. This was done as follows. For the same days where experimental antibody titers exist, the numerical results were estimated. Then we computed the GMT of the numerical values found for these days. For example, suppose that a given group (30–45 days) has five individuals with antibody titers obtained on days 32, 35, 41, 43, and 45. The geometric mean was computed using the levels of antibodies estimated by the model in those same days.

## Data Availability

Model parameters and initial conditions used in simulations are included in this published article. Code to solve the mathematical model can be made available upon request to the authors.

## Appendix

### Initial condition and parameters tables

See Tables 11 and 12.

**Table 11:** Model variables and their initial values

Variable	Description	Initial value	Unit
*V*	Vaccine virus	724	copies/mL
$A_p$	Immature APCs	$10^6$	cells/mL
$A_{pm}$	Mature APCs	0	cells/mL
$T_{hn}$	Naïve CD4+ T cells	$10^6$	cells/mL
$T_{he}$	Effector CD4+ T cells	0	cells/mL
$T_{kn}$	Naïve CD8+ T cells	5x $10^5$	cells/mL
$T_{ke}$	Effector CD8+ T cells	0	cells/mL
*B*	B cells	$2.5 \times 10^5$	cells/mL
$P_s$	Short-lived plasma cells	0	cells/mL
$P_l$	Long-lived plasma cells	0	cells/mL
$B_m$	Memory B cells	0	cells/mL
*A*	Antibodies	150	mIU/mL

**Table 12:** Model parameters

Parameter	Unit	Value
$\pi _v$	(day$^{-1}$)	$6.80 \times 10^{-1}$
$c_{v1}$	(day$^{-1}$)	$2.63 \times 10^{0}$
$c_{v2}$	(copies/mL)	$6 \times 10^{-1}$
$k_{v1}$	[day$^{-1}$ (mIU/mL)$^{-1}$]	$4.82 \times 10^{-5}$
$k_{v2}$	[day$^{-1}$ (cells/mL)$^{-1}$]	$7.48 \times 10^{-7}$
$\alpha _{ap}$	(day$^{-1}$)	$2.5 \times 10^{-3}$
$\beta _{ap}$	[day$^{-1}$ (copies/mL) $^{-1}$]	$5.5 \times 10^{-1}$
$c_{ap1}$	(copies/mL)	$8 \times 10^{-1}$
$c_{ap2}$	(copies/mL)	$4 \times 10^{1}$
$\delta _{apm}$	(day$^{-1}$)	$5.38 \times 10^{-1}$
$\alpha _{th}$	(day$^{-1}$)	$2.17 \times 10^{-4}$
$\beta _{th}$	(day$^{-1}$)	$1 \times 10^{-7}$
$\pi _{th}$	(day$^{-1}$)	$1 \times 10^{-8}$
$\delta _{th}$	(day$^{-1}$)	$2.2 \times 10^{-1}$
$\alpha _{tk}$	(day$^{-1}$)	$2.17 \times 10^{-4}$
$\beta _{tk}$	(day$^{-1}$)	$1 \times 10^{-5}$
$\pi _{tk}$	(day$^{-1}$)	$1 \times 10^{-8}$
$\delta _{tk}$	(day$^{-1}$)	$3 \times 10^{-4}$
$\alpha _{b}$	(day$^{-1}$)	$6.0 \times 10^{0}$
$\pi _{b1}$	(day$^{-1}$)	$4.83 \times 10^{-6}$
$\pi _{b2}$	(day$^{-1}$)	$1.27 \times 10^{-8}$
$\beta _{ps}$	(day$^{-1}$)	$6.72 \times 10^{-4}$
$\beta _{pl}$	(day$^{-1}$)	$5.61 \times 10^{-6}$
$\beta _{bm}$	(day$^{-1}$)	$1 \times 10^{-6}$
$\delta _{ps}$	(day$^{-1}$)	$2.0 \times 10^{0}$
$\delta _{pl}$	(day$^{-1}$)	$2.4 \times 10^{-4}$
$\gamma _{bm}$	(day$^{-1}$)	$9.75 \times 10^{-4}$
$\pi _{bm1}$	(day$^{-1}$)	$1 \times 10^{-5}$
$\pi _{bm2}$	(cells/mL)	$2.5 \times 10^{3}$
$\pi _{ps}$	(day$^{-1}$)	$2 \times 10^{-3}$
$\pi _{pl}$	(day$^{-1}$)	$6.8 \times 10^{-4}$
$\delta _a$	(day$^{-1}$)	$4 \times 10^{-2}$

## Abbreviations

YF: Yellow fever
YFV: Yellow fever vaccine
HIV: Human Immunodeficiency Virus
GMT: Geometric mean titers
PRNT: Plaque Reduction Neutralisation Test
NV: Naïve volunteers
PV: Primary vaccinated volunteers
RV: Revaccinated volunteers
DMARDs: Disease-modifying antirheumatic drugs
ODEs: Ordinary differential equations
APCs: Antigen-presenting cells
IDE: Integrated development environment

## Footnotes

1 State of unregulated immune function in the elderly, which contributes to increased susceptibility to infections, cancer and autoimmunity, and reduced vaccine response. Other controversial issues are: (a) the need for a booster dose also for immunocompetent adults, and (b) the lower vaccine dose that is as immunogenic and safe as the current formulation [3].

2 Antibody titer indicates the level of antibodies in a blood sample, defined as the largest dilution of the blood sample with a dilution agent in which an assay, such as ELISA, still produces a positive result [26].

3 Plaque-Forming Unit is a measure of the number of particles, such as viral particles, capable of forming plaques (visible structures formed within a cell culture) per unit of volume.
